# Pre-breeding for diversification of primary gene pool and genetic enhancement of grain legumes

**DOI:** 10.3389/fpls.2013.00309

**Published:** 2013-08-20

**Authors:** Shivali Sharma, H. D. Upadhyaya, R. K. Varshney, C. L. L. Gowda

**Affiliations:** International Crops Research Institute for the Semi-Arid TropicsHyderabad, India

**Keywords:** wild species, germplasm, pre-breeding, genomics, trait-specific

## Abstract

The narrow genetic base of cultivars coupled with low utilization of genetic resources are the major factors limiting grain legume production and productivity globally. Exploitation of new and diverse sources of variation is needed for the genetic enhancement of grain legumes. Wild relatives with enhanced levels of resistance/tolerance to multiple stresses provide important sources of genetic diversity for crop improvement. However, their exploitation for cultivar improvement is limited by cross-incompatibility barriers and linkage drags. Pre-breeding provides a unique opportunity, through the introgression of desirable genes from wild germplasm into genetic backgrounds readily used by the breeders with minimum linkage drag, to overcome this. Pre-breeding activities using promising landraces, wild relatives, and popular cultivars have been initiated at International Crops Research Institute for the Semi-Arid Tropics (ICRISAT) to develop new gene pools in chickpea, pigeonpea, and groundnut with a high frequency of useful genes, wider adaptability, and a broad genetic base. The availability of molecular markers will greatly assist in reducing linkage drags and increasing the efficiency of introgression in pre-breeding programs.

## Introduction

Grain legumes are second in importance to human and animal diets after cereals and occupy an important place in the world's food and nutrition economy. Being the primary source of high quality dietary protein, grain legumes are important in alleviating protein deficiency and malnutrition prevailing among poor people in developing countries, as well as contributing significantly to global food and nutritional security. Further, grain legumes provide high quality nutritious fodder for animal consumption. In addition to food and fodder, the nitrogen fixing capacity of grain legumes decreases the need for direct application of N-fertilizers and makes them an important component in cropping systems for improving and sustaining soil fertility and texture (Graham and Vance, 2003).

From the nutritional viewpoint, grain legumes are usually deficient in sulphur-containing amino acids-methionine, cysteine and tryptophan, but are rich in other essential amino acid, lysine. The reverse is true with cereals (Rockland and Radke, 1981). Therefore, the dietary mixture of cereals with legumes constitutes a source of balanced diet to the poor farmers in semi-arid tropical regions. In the past, the staple cereal crops, especially wheat, rice and maize, have received the highest research priority and considerable yield improvements were made in these crops. In contrast, grain legumes are under researched compared to cereals. Low yield potential coupled with biotic and abiotic stresses has further reduced their cultivation by the farmers. Recently, realizing the significance of grain legumes in improving nutrition and the livelihood of poor farmers, more research is now being carried out for their genetic amelioration by various institutes. The International Crops Research Institute for the Semi-Arid Tropics (ICRISAT), Patancheru, India is working for the genetic improvement of three major grain legumes, i.e., chickpea (*Cicer arietinum* L.), pigeonpea (*Cajanus cajan* Millsp.), and groundnut (*Arachis hypogaea* L.).

Grain legumes are cultivated mostly in marginal lands under rainfed conditions, with low and unstable productivity (Kumar and van Rheenen, 2000). Their production is adversely affected by several biotic and abiotic stresses. *Ascochyta* blight, *Botrytis* gray mold, *Fusarium* wilt, and dry root rot in chickpea; *Phytophthora* blight, and sterility mosaic disease in pigeonpea; and early and late leaf spots (LLSs), rust and aflatoxin contamination in groundnut are the most important and widely distributed diseases affecting yield and quality. Besides, pod borer (*Helicoverpa armigera* L.), drought, heat, salinity, water-logging are the other important stresses potentially limiting their productivity worldwide. Grain legumes, including chickpea, pigeonpea and groundnut have a narrow genetic base, due to the bottlenecks associated with their evolution and domestication, as well as due to the replacement of locally adapted crop landraces by the genetically advanced modern varieties. Low grain legume productivity due to biotic/abiotic stresses coupled with limited genetic variation in the cultivated gene pool necessitates the identification and utilization of diverse germplasm sources to develop new high-yielding cultivars with a broad genetic base.

## Grain legumes genetic resources

Plant genetic resources are reservoirs of natural genetic variation and provide raw material for crop improvement programs. About 7.4 million germplasm accessions of different crops have been collected and/or assembled and conserved in over 1750 *ex situ* genebanks worldwide (FAO, 2010) and the grain legumes constitute the second largest collection (15%) after cereals (45%). Globally, ~1.1 million grain legume germplasm accessions are conserved in different genebanks, of which ICRISAT genebank holds ~50,000 accessions including cultivated and wild relatives of chickpea (20,267 accessions), pigeonpea (13,771 accessions), and groundnut (15,445 accessions) from 133 countries. Besides ICRISAT, other major genebanks holding grain legume germplasm are the National Bureau of Plant Genetic Resources (NBPGR), New Delhi, India (16,881 *Cicer*, 12,900 *Cajanus* and 14,593 *Arachis* accessions); the International Centre for Agricultural Research in Dry Areas (ICARDA), Aleppo, Syria (13,818 *Cicer* accessions); the United States Department of Agriculture (USDA), USA (9964 *Arachis* accessions); and the Directorate of Groundnut Research (DGR), Junagadh, India (8934 *Arachis* accessions) (updated report based on species in http://apps3.fao.org/wiews/germplasm_query.htm?i_l=EN).

At present, emphasis is on conserving accessions safely with duplication both within and outside the CGIAR. At global level, the Svalbard Global Seed Vault has been established in the Norwegian island of Spitsbergen to store seed samples of a wide variety of plant species in an underground cavern. This seed vault will provide an insurance against the loss of seeds in genebanks, as well as a refuge for seeds in the case of large-scale regional or global crises. Over the last 5 years, >780,000 seed samples belonging to about 840 genera have been deposited in this vault of which about 170,000 accessions belong to 19 legume genera/species (http://www.nordgen.org/sgsv/index.php?app=data_unit&unit= sgsv_by_species&PHPSESSID=1hoc3n9kkdmng79o5vlqmsgs25).

## Use of germplasm in grain legume improvement

Surprisingly, in spite of large collections, only a few germplasm accessions (<1%) have been utilized in crop improvement programs such as in wheat (Dalrymple, 1986), maize (Cantrell et al., 1996), spring barley (Vellve, 1992), soybean (Mikel et al., 2010), and other grain legumes (Kumar et al., 2004). India has one of the largest grain legume breeding programs and has released about 230 cultivars of chickpea, pigeonpea, lentil, black gram, and green gram through hybridization and selection (data up to 2003). Pedigree analysis of these cultivars revealed that Pb-7 in chickpea, T-1 and T-190 in pigeonpea, L-9-12 in lentil, T-9 in black gram, and T-1 in green gram were the most frequently used parents (Kumar et al., 2004). In ICRISAT, which has the largest germplasm collections of chickpea, pigeonpea and groundnut, the breeding programs have used only 91 chickpea (0.4% of total 20,267 accessions), 54 pigeonpea (0.4% of total 13,771 accessions) and 171 groundnut (1.1% of total 15,445 accessions) germplasm accessions to develop advanced breeding lines. Further, the increase in accession numbers in genebanks and lack of corresponding increase in their utilization by the crop improvement scientists also indicate that the collections are not being utilized to their full potential (Marshall, 1989). As a global responsibility, ICRISAT's genebank has supplied over 303,000 samples of grain legumes, which includes 131,924 samples of chickpea, 71,826 samples of pigeonpea, and 99,325 samples of groundnut accessions to scientists across 136 countries. Besides this, over 370,000 samples of these legumes have also been distributed to researchers within ICRISAT during 1974–June 2013. Some of the germplasm accessions supplied from ICRISAT genebank have been released directly as varieties by NARS such as 15 chickpea germplasm accessions released as varieties in 15 countries, 10 pigeonpea germplasm accessions in seven countries and 11 groundnut germplasm accessions in 15 countries (Table 1). These varieties have greatly benefited the farmers by contributing to increase in production and productivity in these countries. However, the pattern of demand and consequent supply has shown a greater demand for a few specific germplasm accessions. In a period of 35 years, in chickpea, two accessions, ICC 4918 and ICC 4973 were supplied over 350 times, and four accessions more than 200 times. In pigeonpea, one accession, ICP 7035 was supplied 330 times, and seven accessions more than 200 times. Similarly, in groundnut, one accession, ICG 799 was supplied over 300 times, and five accessions about 200 times. Further, 3740 chickpea, 3088 pigeonpea and 1018 groundnut accessions have not been requested at all, while 7339 chickpea, 7001 pigeonpea and 7582 groundnut accessions have been supplied less than five times (as on 30 May 2013).

**Table 1:** **List of chickpea, pigeonpea and groundnut germplasm accessions directly released as varieties for cultivation**.

**Accession number**	**Country of origin**	**Year of release**	**Country of release**	**Released name**
**CHICKPEA**				
ICC 237	India	1988	Oman	ICC 237
ICC 552	India	–	Myanmar	Yezin 1
ICC 3274	Iran	1999	Bangladesh	Bari Chhola7
ICC 4923	India	1978	India	Jyothi
ICC 4944	India	–	Myanmar	Keyhman
ICC 4951	India	–	Myanmar	ICC 4951
ICC 4998	India	1994	Bangladesh	Bina-Sola 2
ICC 6098	India	1987	Nepal	Radha
ICC 8521	Italy	–	USA	Aztee
ICC 8649	Afghanistan	1987	Sudan	Shendi
ICC 11879	Turkey	1986	Turkey	–
	Turkey	1982	Syria	Ghab 1
	Turkey	1987	Morocco	–
	Turkey	1988	Algeria	–
ICC 13816	USSR (former)	1986	Syria	Ghab 2
	USSR (former)	1984	Algeria	Yialousa
	USSR (former)	–	Cyprus	–
	USSR (former)	1987	Italy	Sultano
ICC 14808	India	2006	Ethiopia	Yelbey
ICC 14880	India	1997	Australia	Hira
ICC 14911	USSR (former)	1987	Morocco	–
	USSR (former)	1986	Turkey	–
**PIGEONPEA**				
ICP 6997	India	1992	Nepal	Rampur Rhar
ICP 7035	India	1989	Philippines	–
	India	2005	India	JK Sweety (JKPL 5)
	India	2003	China	Guimu 4
	India	1985	Fiji	Kamica
ICP 8863	India	1985	India	Maruti
ICP 9145	Kenya	1988	Malawi	Nandolo wa nswana
ICP 9905	India	1991	Venezuela	La Cerrera
ICP 11384	Nepal	1992	Nepal	Bageswari
ICP 11916	India	1991	Venezuela	Aroa
ICP 13092	Kenya	2005	India	JK Sixer (JKPL 6)
ICP 13829	Granada	1991	Venezuela	Cerro Pelon
ICP 14770	India	1989	India	Abhaya
**GROUNDNUT**				
ICG 221	India	1994	Swaziland	–
ICG 273	Argentina	1994	Ethiopia	Sedi
ICG 1697	Peru	1998	Indonesia	Singa
ICG 1703	Peru	1998	Indonesia	Panter
	Peru	2003	Thailand	Kalasin 2
ICG 2271	USA	–	Nepal	–
ICG 2974	Israel	1984	Myanmar	Sinpadetha 3
	Israel	1985	Tanzania	Johri
ICG 7794	USA	1989	Ethiopia	–
ICG 7827	India	1992	Philippines	UPL Pn 10
	India	1984	Myanmar	Sinpadetha 2
ICG 7878	India	–	Mali	Waliyartiga
	India	1990	Mauritius	ICG 7878
	India	2011	Mozambique	JL24
ICG 7886	Peru	1987	Jamica	Cardi-Payne
ICG 12991	India	2004	Zambia	Msandile
	India	2001	Malawi	Baka
	India	2002	Uganda	Serenut 4T
	India	2002	Mozambique	Nametil

## Reasons for low use of germplasm

**Figure 1:**
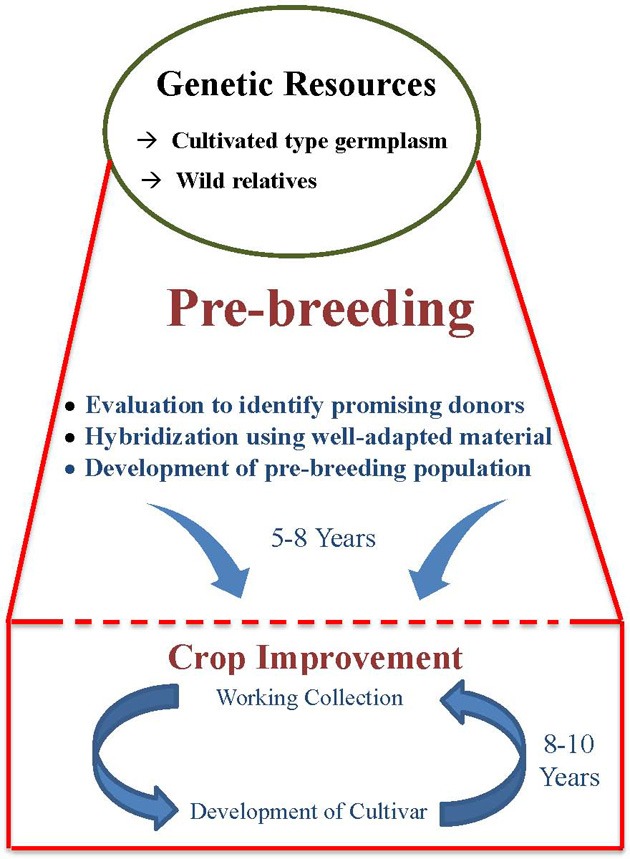
**Pre-breeding as a bridge between genetic resources and crop improvement**.

The factors responsible for low use of germplasm in crop improvement programs are the large size of germplasm collections, the breeders' preference for working collections, and the linkage drag associated with utilizing wild relatives in crop improvement programs. Large germplasm collections of most crops, including grain legumes, poses problem for their meaningful multi-locational evaluation. Thus, there is a lack of information on traits of economic importance, which often shows high genotype × environment interaction, for large number of germplasm accessions. This creates a problem for the breeders to select the appropriate genetic diversity for use in their breeding programs. However, strategies are now available to overcome the problems associated with the large size of germplasm collections. This includes the development of representative small-sized subsets such as core (Frankel and Brown, 1984) and mini core (Upadhyaya and Ortiz, 2001) collections that contain the majority of allelic diversity. Besides large size, there are apprehensions among breeders about poor adaptability of germplasm and linkage drag. Linkage drag is the most important factor responsible for low use of germplasm in crop improvement and is the major reason for the need for pre-breeding. While using unknown and wild germplasm, comparatively more efforts, time and resources are required to break undesirable linkage drag during the development process, particularly for regional adaptability to climates, crop management, biotic and abiotic stresses, and overall agronomic performance. This makes the breeding program comparatively more lengthy and cumbersome (Figure 1). For these reasons, breeders use their working collection in breeding programs which results in re-circulation of same genotype and hence narrow genetic base of the released cultivars. Nevertheless, the above reservations and over cautiousness toward linkage drag has resulted in continuous narrowing down of genetic variability among modern cultivars, with implications for survival from several biotic and abiotic stresses.

## Pre-breeding for accessing novel genes

The success of any crop improvement program depends on the availability of sufficient genetic variability, but this variability must be in conventionally usable form. The variability available in any crop germplasm conserved in genebanks for present and future use belongs broadly to the following three groups:

1. Cultivated type
2. Cross-compatible wild type
3. Cross-incompatible wild type

The genetic variability in cultivated type germplasm is either in poor agronomic background or in genetic background not adapted to the breeding or target climate for its direct use in conventional breeding programmes. The exploitation of genetic variability in wild species for cultivar improvement is hindered mainly by linkage drag and different incompatibility barriers between cultivated and wild species. Under such situations, pre-breeding offers a unique tool to enhance the use of genetic variability present both in cultivated and wild type germplasm.

Pre-breeding involves all the activities associated with identification of desirable traits and/or genes from unadapted germplasm (donor) that cannot be used directly in breeding populations (exotic/wild species), and to transfer these traits into well-adapted genetic backgrounds (recipients) resulting in the development of an intermediate set of material which can be used readily by the plant breeders in specific breeding programmes to develop new varieties with a broad genetic base (Figure 1).

### Identification of trait-specific germplasm accessions

#### Cultivated type germplasm

Enormous efforts are needed to evaluate germplasm for traits of economic importance, as well as to screen for biotic and abiotic stress related traits through reliable and standardized techniques for identifying potential donors. Owing to the large size of germplasm collections, this becomes a costly and resource-demanding task. Following the concept of Frankel and Brown (1984) for sampling diversity, a number of small-sized subsets such as core (10% of the entire collection in size) and mini core (10% of core or 1% of entire collection) collections representing the diversity of entire collections have been developed in chickpea, pigeonpea and groundnut (Table 2). Due to their small size, these collections, especially mini core collections, provide an easy means to evaluate these collections across multilocations and to identify promising germplasm accessions as new and potential donors for various traits. Over 90 sets of mini core collections of SAT legumes have been shared by ICRISAT genebank with researchers in 20 countries. Evaluation of germplasm including core/mini core collections globally has resulted in the identification of new and diverse trait-specific germplasm accessions for agronomic and nutritional traits, as well as for resistance/tolerance to various biotic/abiotic stresses (reviewed in Upadhyaya et al., 2010). For example, new and promising sources for early maturity, large seed size, yield and component traits, and for nutrition-related traits, are now available for improvement of chickpea, pigeonpea and groundnut (Table 3). Similarly, for abiotic/biotic stresses, new sources of tolerance/resistance have been identified in chickpea, pigeonpea and groundnut (Table 4). For example, in chickpea, in addition to the well-known drought tolerant accession, ICC 4958, new sources of drought tolerance have been identified using different techniques (Table 4); ICC 13124 has the highest drought tolerance efficiency (DTE), lowest drought susceptibility index (DSI) and the highest harvest index (HI) and has been identified as the most drought tolerant accession (Parameshwarappa and Salimath, 2008; Parameshwarappa et al., 2010). Recently, to combat the effect of climate change on grain legume production, new and diverse sources of heat tolerance (Krishnamurthy et al., 2011a; Upadhyaya et al., 2011a), herbicide tolerance (Tar'an et al., 2010) and accessions with greater biological nitrogen fixation (BNF) capacity (Biabani et al., 2011) for chickpea and water-logging tolerant accessions for pigeonpea improvement (Krishnamurthy et al., 2011b) (Table 4) have also been identified.

**Table 2:** **Core and mini core collections as reported in chickpea, pigeonpea and groundnut**.

**Reduced subset**	**Accessions used (No.)**	**Collection**	**Accessions in constituted**	**References**
			**collection (No.)**	
Chickpea	16,991	Core	1956	Upadhyaya et al., 2001a
	3350	Core	505	Hannan et al., 1994
	1002	Core	158	Kibret, 2012
	–	*Kabuli* core	103	Pouresmael et al., 2009
	1956	Mini core	211	Upadhyaya and Ortiz, 2001
	482	Mini core	39	Biabani et al., 2011
Pigeonpea	12,153	Core	1290	Reddy et al., 2005
	1290	Mini core	146	Upadhyaya et al., 2006a
Groundnut	630	Valencia core	77	Dwivedi et al., 2008b
	7432	Core	831	Holbrook et al., 1993
	6390	Core	576	Jiang et al., 2008
	4738	Asian core	504	Upadhyaya et al., 2001b
	14,310	Core	1704	Upadhyaya et al., 2003
	831	Mini core	111	Holbrook and Dong, 2005
	1704	Mini core	184	Upadhyaya et al., 2002

**Table 3:** **Promising germplasm accessions for agronomic and nutrition-related traits in chickpea, pigeonpea and groundnut**.

**Traits**	**Chickpea**	**Pigeonpea**	**Groundnut**
Early maturity	28 accession (ICC# 16641, 16644, 11040, 11180, and 12424; Upadhyaya et al., 2007a)^*^	8 accession (ICP# 14900, 1156, 14471, 14903, and 16309; Upadhyaya et al., 2010)	21 accession (ICG# 4558, 4890, 9930, 11605, and 5512; Upadhyaya et al., 2006b)
Large seed	11 accessions (ICC# 14190, 14194, 7345, 17452, 19189, and 17109; Gowda et al., 2011)	3 accessions (ICP# 14976, 13359, and 13139; Upadhyaya et al., 2010)	12 accessions (ICG# 2381, 5016, 5051, 5745, and 5662; Upadhyaya et al., 2010)
	4 accessions (ICC# 14199, 14197, 14203, and 12033; Kaul et al., 2007)		
	9 accessions (ICC# 17457, 17452, 19189, 17456, and 18591; Kashiwagi et al., 2007)		
Yield and component traits	39 accessions (ICC# 6122, 8474, 8155, 12034, and 4871; Upadhyaya et al., 2007b)	4 accessions (ICP# 8860, 11230, 4167, and 8602; Upadhyaya et al., 2010)	60 accessions (ICG# 4, 29, 3443, 14161, 11188, 7140, and 2918; Upadhyaya et al., 2005)
	8 accessions (ICC# 13124, 12654, 9848, 6279, 5879, and 10341; Parameshwarappa et al., 2011a,b)		
	6 accessions (ICC# 14778, 6279, 4567, 4533, 1397, and 12328; Meena et al., 2010)		
Protein content (>30%)	–	6 accessions (ICP# 4575, 7426, 8266, 11823, 12515 and 12680; Upadhyaya et al., 2013)	18 accessions (ICG # 36, 5779, 3421, 3584, and 2019; Upadhyaya et al., 2012b)
Oil content (>50%)	–	–	18 accessions (ICG # 442, 397, 5779, 4955 and 14710; Upadhyaya et al., 2012b)
Oleic acid (≥60%)	–	–	18 accessions (ICG # 6022, 12625 and 11088 in subsp. *fastigiata*; and ICG# 2381, 10185, 15419, 12276, 7243 and 6766 in subsp. *hypogaea*; Upadhyaya et al., 2012b)
Oleic/Linoleic acid ratio (>3.0)	–	–	18 accessions (ICG # 6022, 12625 and 1274 in subsp. *fastigiata*; and ICG# 2381, 10185, 15419, 12276, 7243 and 6766 in subsp. *hypogaea*; Upadhyaya et al., 2012b)

* A few promising accessions are given in parenthesis with reference.

**Table 4:** **New sources of tolerance/resistance to abiotic/biotic stresses for chickpea, pigeonpea and groundnut improvement**.

**Traits**	**Promising germplasm**	**References**
**CHICKPEA**		
Drought	Root length density: 9 accessions (ICC# 8261, 10885, 16796, 13816 and 13599)^*^	Kashiwagi et al., 2005
	Root depth: 9 accessions (ICC# 3512, 15697, 13523, 1356, and 4872)	Kashiwagi et al., 2005
	SPAD Chlorophyll Meter Reading (SCMR): 4 accessions (ICC# 16374, 1422, 16903, and 10945)	Kashiwagi et al., 2006, 2010
	Canopy temperature: 3 accessions (ICC# 3325, 867, and 14799)	Kashiwagi et al., 2008
	Terminal drought: 5 accessions (ICC# 867, 1923, 9586, 12947 and 14778)	Krishnamurthy et al., 2010
Salinity	10 accessions (ICC# 10755, 13124, 13357, 15406, and 15697)	Serraj et al., 2004
	15 accessions (5003, 15610, 1431, 4593, and 12155)	Vadez et al., 2007
	12 accessions (9942, 6279, 11121, 456 and 14799)	Krishnamurthy et al., 2011c
Heat	18 accessions (ICC# 456, 637, 1205, 3362, and 3761)	Krishnamurthy et al., 2011a
	10 accessions (14346, 5829, 6121, 13124 and 14284)	Upadhyaya et al., 2011a
Herbicide (imazethapyr and imazamox)	4 accessions (ICC# 2242, 2580, 3325 and ILC 4279)	Tar'an et al., 2010
Multiple disease	18 accessions (ICC# 1710, 2242, 2990, 11284, and 12328)	Pande et al., 2006
**PIGEONPEA**		
Water-logging	24 accessions (ICP# 1279, 7057, 7148, 10397, and 16309)	Krishnamurthy et al., 2011b
Salinity	16 accessions (ICP# 8860, 7426, 14722, 11477, and 6128)	Srivastava et al., 2006
Combined resistance for *Fusarium* wilt and sterility mosaic	6 accessions (ICP# 6739, 8860, 11015, 13304, 14638, and 14819)	Sharma et al., 2012
**GROUNDNUT**		
Drought	18 accessions (ICG# 14523, 6766, 7243, 862, and 6654)	Upadhyaya, 2005
	30 accessions (11088, 12697, 8751, 3140, and 3584)	Hamidou et al., 2011
Low temperature	15 accessions (ICG# 12625, 7898, 11130, 6148, and 7013)	Upadhyaya et al., 2009
Salinity	14 accessions (ICG# 5195, 442, 7283, 1711, and 2106)	Srivastava, 2010
*Rhizoctonia* limb rot resistant	6 accessions (PI# 343398, 343361, 288178, 331326, 497351, and 274193)	Franke et al., 1999
Late leaf spot	7 accessions (ICG# 12625, 11426, 12672, 13787, 14475, 2857, and 8760)	Kusuma et al., 2007
Rust	22 accessions (ICG 9809, 11088, 11426, 13787, and 8760)	Kusuma et al., 2007
*A. flavus*	6 accessions (ICG# 14985, 3673, 6025, 12625, 13787, and 8760),	Kusuma et al., 2007
	20 accessions (6813, 12370, 4750, 4156, and 12697)	Jiang et al., 2010
Bud necrosis	4 accessions (ICG# 875, 928, 1668, and 14466)	Ahmed, 2008
Combined resistance to *Sclerotinia* blight, pepper spot and web blotch	5 accessions (PI# 274193, 497599, 458619, 468195, and 259796)	Damicone et al., 2010

* A few promising accessions are given in parenthesis.

#### Wild type germplasm

Wild species are the reservoir of many useful genes/alleles as they have evolved under natural selection to survive climate extremes. Wild species of *Cicer, Cajanus*, and *Arachis* have been extensively screened and several of them were reported to have very high level of resistance/tolerance to various stresses. Among wild *Cicer* species, *C. bijugum, C. judaicum*, and *C. pinnatifidum* are the most important sources having the highest levels of resistance/tolerance to multiple stresses (reviewed in Gaur et al., 2009; Sharma et al., 2013). Wild *Cajanus* species especially, *C. scarabaeoides, C. acutifolius, C. platycarpus, C. reticulatus, C. sericeus*, and *C. albicans* provide resistance to pod borer, *Helicoverpa armigera* (Rao et al., 2003; Sujana et al., 2008; Sharma et al., 2009). Evaluation of wild *Cajanus* species has identified new sources of resistance to *Alternaria* blight, *Phytophthora* blight, sterility mosaic virus, pod fly and nematodes and tolerance to salinity, drought, and photoperiod insensitivity (reviewed in Upadhyaya et al., 2013). Good sources of resistance for bruchids (*Callosobrochus maculatus*) have also been identified in *C. scarabaeoides*, *C. acutifolius*, and *C. platycarpus* (Jadhav et al., 2012). Similarly, wild *Arachis* species harbor very high levels of resistance/tolerance to many biotic/abiotic stresses (reviewed in Dwivedi et al., 2008a; Upadhyaya et al., 2012a). These wild species are the potential donors to develop genome-wide introgression (GWI) lines for the genetic amelioration of chickpea, pigeonpea and groundnut.

### Pre-breeding for grain legume genetic enhancement

The success of any pre-breeding program depends mainly upon three factors: (1) identification of promising donor with good expression of the trait; (2) type of germplasm (Cultivated/cross-compatible wild type/cross-incompatible wild type); and (3) agronomic performance of the donors. New and diverse sources of variation for agronomic and nutrition-related traits and resistant/tolerant sources for biotic/abiotic stresses are now available both in cultivated and wild type germplasm and can be utilized to develop new pre-breeding populations having greater variability for various traits. In the past, a few promising wild type accessions have been utilized by some researchers for the improvement of chickpea, pigeonpea and groundnut and are given hereunder.

#### Pre-breeding for biotic/abiotic stresses

***Chickpea.*** Of the eight annual wild *Cicer* species, only *C. reticulatum* is readily crossable with cultivated chickpea resulting in fertile hybrid. The exploitation of the remaining seven annual wild *Cicer* species requires specialized techniques such as the application of growth hormones, embryo rescue, ovule culture, and tissue culture techniques (Badami et al., 1997; Mallikarjuna, 1999; Lulsdorf et al., 2005; Mallikarjuna and Jadhav, 2008). Utilization of *C. reticulatum* accession, ILWC 119 in crossing programme has resulted in the development of two cyst nematode resistant chickpea germplasm lines ILC 10765 and ILC 10766 (Malhotra et al., 2002). Beneficial traits such as cold tolerance and a high degree of resistance to wilt, foot rot, root rot, and *Botrytis* gray mold have also been introgressed from *C. reticulatum* and *C. echinospermum* into cultivated chickpea (ICARDA, 1995; Singh et al., 2005; Ramgopal et al., 2012). Using novel techniques, interspecific hybrids have been produced between *C. arietinum* × *C. judaicum* (Verma et al., 1990; Verma and RaviSandhu, 1995; Singh et al., 1999), *C. arietinum* × *C. pinnatifidum* (Verma et al., 1990; Badami et al., 1997; Mallikarjuna, 1999; Mallikarjuna and Jadhav, 2008), *C. arietinum* × *C. cuneatum* (Singh and Singh, 1989), and *C. arietinum* × *C. bijugum* (Verma et al., 1990; Singh et al., 1999; Mallikarjuna et al., 2007) to introgress desirable alien genes from these cross-incompatible wild *Cicer* species into cultivated chickpea. These interspecific hybrids have contributed significantly toward the development of genomic resources for chickpea improvement.

***Pigeonpea.*** Wild *Cajanus* species, especially the cross-compatible secondary gene pool species, have been used for the genetic improvement of pigeonpea and the most significant achievement includes the development of unique cytoplasmic-nuclear male sterility system (CMS). The CMS systems have been developed with cytoplasm derived from wild *Cajanus* species namely *C. sericeus* (Ariyanayagam et al., 1995), *C. scarabaeoides* (Tikka et al., 1997; Saxena and Kumar, 2003), *C. volubilis* (Wanjari et al., 2001), *C. cajanifolius* (Saxena et al., 2005), *C. lineatus* (Saxena et al., 2010), and *C. platycarpus* (Mallikarjuna et al., 2011a). For pigeonpea improvement, the potential for genetic introgression of salinity tolerance from wild relative, *Atylosia albicans* to cultivated pigeonpea has been demonstrated in the *F*_1_ (Subbarao et al., 1990). Further, utilization of *C. acutifolius* as the pollen parent has resulted in the development of advanced generation population having resistance to pod borer (Mallikarjuna et al., 2007), variation in seed color and high seed weight. For *Phytophthora* blight, resistant genes have been transferred from *C. platycarpus* into cultivated pigeonpea and the resistant plants identified at seedling stage showed resistance across all stages of their life cycle (Mallikarjuna et al., 2005).

***Groundnut.*** Utilization of wild *Arachis* species following interspecific hybridization has resulted in the development of many elite germplasm lines and cultivars with improved level of resistance to diseases and insect-pests. At ICRISAT, several elite lines have been developed with desirable characters transferred from wild *Arachis* species such as ICGV 86699 (Reddy et al., 1996) with multiple pest resistance, ICGV 87165 (Moss et al., 1998) with multiple disease and insect resistance, ICGV 99001 and 99004 with resistance to LLS, and ICGV 99003, and 99005 resistant to rust (Singh et al., 2003). Varieties such as Spancross (Hammons, 1970), Tamnut 74 (Simpson and Smith, 1975), Coan (Simpson and Starr, 2001), NemaTAM (Simpson et al., 2003), ICGV-SM 85048 (Nigam et al., 1998), and ICGV-SM 86715 (Moss et al., 1998), having a genetic base from wild *Arachis* species, were released for cultivation, mostly in USA. The development and utilization of synthetic amphiploids such as TxAG-6 with large genetic variation (Simpson et al., 1993) has made possible the transfer of resistance genes from wild species into cultivated groundnut. TxAG-6 is a synthetic amphiploid derived from crossing an AA-genome donor hybrid (*A. cardenasii* × *A. diogoi*) with a BB genome species, *A. batizocoi*, followed by colchicine treatment of the sterile triploid to produce fertile hexaploid, TxAG-6 (Simpson et al., 1993). This amphiploid has been synthesized using species that are not in the direct lineage of the cultivated groundnut. However, it is crossable with the cultivated groundnut and produced fertile progenies, thus proving useful for introducing genetic variability into the cultigen. Using this amphiploid in crossing programs with cultivated groundnut has resulted in the release of two cultivars, Coan and NemaTAM, carrying genes for root-knot nematode (*M. arenaria*) resistance from *A. cardenasii* (Simpson and Starr, 2001; Simpson et al., 2003).

#### Pre-breeding for improvement of agronomic and nutrition-related traits

Besides resistant sources, studies also indicated the possibility of improving agronomic traits, including yield, through introgression of genes from the wild species into the cultigens. In chickpea, by hybridizing *C. reticulatum* and *C. echinospermum* with cultigen, Singh and Ocampo (1997) generated high-yielding recombinant lines without any known undesirable traits from the wild species. In a similar study, introgressing wild genes from *C. reticulatum* into cultivated chickpea generated nine genotypes with high yield potential, resistance to soil-borne diseases and adaptation to water-limited environments. Three lines BG 1100, BG 1101, and BG 1103 produced 20% higher seed yield than the best-adapted cultivars and were resistant to *Fusarium* wilt (*Fusarium oxysporum* f.sp. *ciceris*) (Yadav et al., 2004). Interspecific derivatives possessing a high degree of resistance to diseases such as wilt, root rot and foot rot, and high yield, have been obtained from *C. arietinum* × *C. reticulatum* crosses (Singh et al., 2005). In ICRISAT, using two *C. reticulatum* accessions (110–113 days to 50% flowering, 143–150 days to maturity, 12–16 g 100-seed weight) in a hybridization program, several progenies were selected which took 8–21 days less to flower, 6–33 days less to mature, with 20–103% larger seeds, and 97–217% greater seed yield than respective cultivated (*C. arietinum*) parent (Upadhyaya, 2008). From *C. arietinum* × *C. judaicum* cross, a pre-breeding line IPC 71 having high number of primary branches, more pods per plant and green seeds has been developed for use in chickpea improvement programs (Chaturvedi and Nadarajan, 2010).

**Figure 2:**
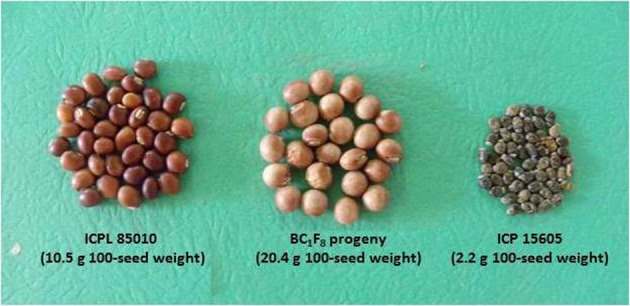
**Improvement of seed traits in cultivated pigeonpea by using *Cajanus acutifolius***.

Further, utilization of wild *Cajanus* species has also contributed significantly toward the improvement of agronomic performance and nutritional quality of cultivated pigeonpea. A high protein line, ICPL 87162 was developed from the cross *C. cajan* × *C. scarabaeoides* (Reddy et al., 1997). This line contains 30–34% protein content compared to control cultivar (23% protein). Breeding lines with high protein content have also been developed from *C. sericeus*, *C. albicans*, and *C. scarabaeoides*. Utilization of wild *Cajanus* species has resulted in the development of several lines such as HPL 2, HPL 7, HPL 40, and HPL 51 having high protein and seed weight (Saxena et al., 1987). Recently, scientists at ICRISAT have generated segregants with up to 20.4 g 100-seed weight from the cross between cultivated pigeonpea, ICPL 85010 (10.5 g 100-seed weight) and *C. acutifolius*, ICP 15605 (2.2 g 100-seed weight) (Figure 2).

Similarly in groundnut, by using an amphiploid TxAG-6 [(*Arachis cardenasii* × *A. diogoi*) × *A. batizocoi*] with very low 100-seed weight (~12 g) and poor pod yield (2–5 g plant^−1^), breeding lines have been developed. These have higher 100-seed weight (up to 95 g) compared to 32 g of control cultivar TMV 2 and 23–68% more pod yields than TMV 2 (3343 kg ha^−1^). They also have 10–50% more pod yield than the high yielding control cultivar ICGV 91114 (3741 kg ha^−1^, 49 g 100-seed weight^−1^) (Upadhyaya, 2008). This demonstrates that the novel alleles of wild relatives that were considered to be lost in evolution to cultivated types could still be used to enhance the important agronomic and nutrition-related traits in cultivars.

## Pre-breeding for climate smart grain legume crops

The emerging threat to global crop production is climate variability, leading to frequent droughts as a result of erratic rainfall, high temperature prevalence, water-logging, increased soil salinity, and emergence of new insect-pests and diseases. Due to climate change, several areas are now becoming unfit for cultivation of traditional crops. To cope up with this situation, there is a need to breed new crop cultivars with a broad genetic base capable of withstanding frequent climatic fluctuations. In grain legumes, emerging threats due to climate change involves dry root rot (*Rhizoctonia bataticola*) and *Ascochyta* blight in chickpea and *Phytopthora* and *Alternaria* blight in pigeonpea. Frequent droughts coupled with high temperature during flowering to pod filling stage would result in heavy incidence of dry root rot whereas cooler temperature with high humidity are associated with high incidence of *Ascochyta* blight in chickpea (Pande et al., 2010). In pigeonpea, erratic and heavy rainfall (>300 mm in 6–7 days) leading to temporary flooding has resulted in an outbreak of *Phytophthora* blight in Deccan Plateau of India (Sharma et al., 2006). Recently, *Alternaria* blight of pigeonpea is becoming a serious threat in semi-arid tropics due to the untimely rainfall. Changing climatic conditions resulting in high rainfall will favor foliar diseases and some soilborne pathogens such as *Phytophthora, Pythium, Rhizoctonia solani*, and *Sclerotium rolfsii* whereas low rainfall resulting in decreased moisture will lead to the high incidence of *Fusarium* wilt, dry root rot etc. in cool season grain legumes (Pande and Sharma, 2011). Under such situations, pre-breeding activities utilizing wild species demand the highest priority and must focus on the identification of new traits better suited to the new environment for developing climate-resilient cultivars with a broad genetic base.

## Genomic resources for use in pre-breeding for grain legume improvement

Significant progress has been made in the development of large-scale genomic resources in the last decade for all the three SAT legume crops. Further, coordinated efforts have led to the development of large-scale genomic resources such as molecular markers, construction of comprehensive genetic maps, dense consensus maps and identification of marker-trait associations. Some recent reviews provide in-depth detail on quantity, source and development methods of these resources in these three legumes (see reviews Upadhyaya et al., 2011b; Varshney et al., 2012a, 2013a). Simple sequence repeat (SSR) or microsatellite and single nucleotide polymorphism (SNP) markers are the preferred marker systems especially for genetics and breeding applications. In addition, other genotyping systems such as diversity array technology (DArT) have also become available. A total of ~13,000–309,052 SSRs markers have become available in chickpea, pigeonpea and groundnut. ICRISAT, in collaboration with DArT Pty Ltd Australia, has developed DArT arrays with 15,360 features for chickpea (Thudi et al., 2011), groundnut and pigeonpea crops (see Varshney et al., 2013a). Although these DArT arrays showed low polymorphism among genotypes of the primary gene pool, they are of great help in monitoring the alien genome introgression in the cultivated species, as been observed for pigeonpea (Mallikarjuna et al., 2011b).

SNP markers are now gaining more importance due to higher abundance and amenability to high-throughput genotyping. Thousands of SNPs were identified using a variety of approaches in chickpea by Hiremath et al. (2011) (26,082 potential SNPs); in pigeonpea by Dubey et al. (2011) (12,141 SNPs) and Varshney et al. (2012a) (28,104 novel SNPs); and in groundnut by University of Georgia (8478 SNPs) (Nagy et al., 2012).

In addition to development of genomic tools essential for breeding applications, there is a great need to develop cost-effective genotyping platforms/assays. A range of cost-effective SNP genotyping platforms have become available such as Illumina GoldenGate assays for genotyping 768 SNPs in chickpea by University of California-Davis, USA and 1536-SNPs for groundnut by University of Georgia, USA. In order to provide better genotyping deal for small to medium number of samples (<500), VeraCode assays have been developed for 96-plex and 48-plex SNP sets for chickpea and pigeonpea, respectively. Similarly, an alternative genotyping assay (KASPar assay) has been developed by KBiosciences (www.kbioscience.co.uk), which provides flexibility to genotype any number of samples with any number of SNPs. Thus, ICRISAT has developed KASPar assays for 2005 SNPs in chickpea (Hiremath et al., 2012), 1616 SNPs in pigeonpea (Saxena et al., 2012) and 90 SNPs in groundnut (Khera et al., 2013). Besides above mentioned genomic resources and genotyping platforms, draft genome sequences have become available in pigeonpea (Varshney et al., 2012b) and chickpea (Varshney et al., 2013b), while similar efforts are underway in groundnut. The recent availability of the above resources for these three crops has now changed their status from “genomics poor” to “genomics rich” crops.

## Pre-breeding: present status and future outlook

Due to the limited genetic variability available in cultivated germplasm, pre-breeding is gaining importance in most crop improvement programs including wheat (Valkoun, 2001), maize (Nass and Paterniani, 2000), and common bean (Acosta-Gallegos et al., 2007). Recently, under the ICAR-ICARDA collaborative research, efforts are in progress for the improvement of bread wheat, kabuli chickpea, barley, and lentil through pre-breeding and genetic enhancement. At ICRISAT, pre-breeding activities in chickpea, groundnut, and pigeonpea are in progress using promising exotic landraces and wild species as donors along with popular varieties as recipients. The aim is to develop new gene pools with a higher frequency of useful genes, wider adaptability, and a broader genetic base for important agronomic and nutrition-related traits, as well as for resistance/tolerance to important biotic/abiotic stresses. Emphasis is being given to introgress genes for resistance against *Ascochyta* blight, *Botrytis* gray mold, and *Helicoverpa* pod borer in chickpea; for sterility mosaic disease, *Phytophthora* blight, and pod borer in pigeonpea; and for LLS, peanut stem necrosis disease (PSND), and aflatoxin in groundnut. Among the three SAT legumes, cultivated groundnut has the major need for pre-breeding activities due to a narrow gene pool. The tetraploidization event occurred during the evolution of cultivated groundnut has restricted the exchange of genomic regions between wild relatives and cultivated groups. Fortunately, wide crosses resulted in the development of highly diverse autotetraploids and allotetraploids. Simpson et al. (1993) and Fávero et al. (2006) reported development of three amphiploids using a range of wild AA- and BB- genome species such as *A. cardenasii, A. diogoi*, and *A. batizocoi, A. ipaënsis*, *A. duranensis, A. gregoryi*, and *A. linearifolium*. In order to diversify the primary gene pool, new amphiploid and autotetraploid groundnuts have been developed at ICRISAT from diploid hybrids such as *A. magna* × *A. valida, A. magna* × *A. batizocoi, A. batizocoi* × *A. cardenasii, A. batizocoi* × *A. duranensis, A. ipaënsis* × *A. duranensis, A. valida* × *A. duranensis, A. duranensis* × *A. cardenasii, A. kempff mercadoi* × *A. stenosperma, A. diogoi* × *A. cardenasii, A. duranensis* × *A. batizocoi, A. cardenasii* × *A. diogoi, A. duranensis* × *A. valida, A. duranensis* × *A. ipaënsis, A. kempff mercadoi* × *A. hoehnei, A. duranensis* × *A. batizocoi,* and *A. diogoi* × *A. cardenasii* (Mallikarjuna et al., 2011c). Evaluation of a few of these amphiploids has shown resistance to LLS (Mallikarjuna et al., 2012) and PBND (Shilpa et al., 2013). Using wild species of chickpea and pigeonpea, and synthetic amphiploids in groundnut, GWI lines and advanced-backcross (AB) populations are being developed in the genetic background of elite varieties following marker-assisted selection. (For developing AB-population, a selected wild relative is crossed to an elite cultivar followed by two to three backcrosses with targeted elite cultivar to develop segregating BC_2_F_2_ or BC_2_F_3_ populations). Phenotyping and genotyping of these (GWI and AB) populations should help in identifying the lines with enhanced genetic base and minimum linkage drag for use in future breeding programs, as well as to find out the markers associated with traits of interest.

## Initiatives and hope for enriching cultivated gene pool through genomics-assisted pre-breeding

The availability of genomic resources has provided a solution to monitor allele/genome-specific introgression in GWI lines, along with identification of marker-trait association using AB populations. Genomics tools provide efficient tracking for desired and non-desired alien alleles among breeding lines using AB-QTL based breeding approach. Thus, integration of genomic tools with conventional breeding approaches promises to enrich the cultivated gene pool through genomics-assisted pre-breeding. These efforts will help in harnessing the rich diversity of wild relatives possessing superior alleles lost either during domestication or breeding, as well as for exploiting novel alleles. In AB-QTL based breeding approach, genotypic and phenotypic data generated on AB populations are used for identification of QTLs for several economically important agronomic traits. In addition, it also provides an opportunity to select for QTL-near isogenic lines (NILs) in a short time span along with the development of introgression lines with desired features for use in hybridization programmes.

**Figure 3:**
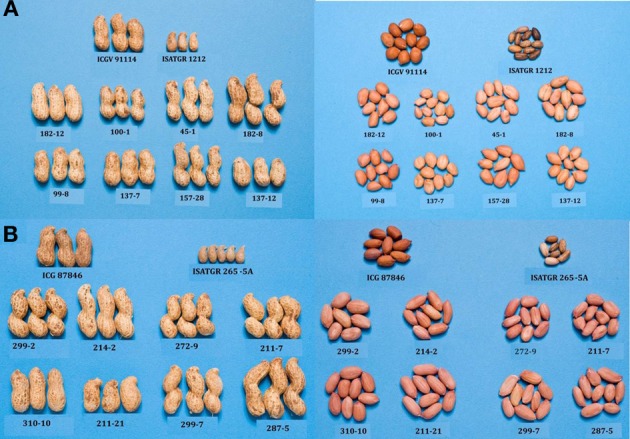
**Variability for pod and seed traits in AB-QTL populations derived from using synthetics in crosses between (A) ICGV 91114 × ISATGR 1212 (above) and (B) ICGV 87846 × ISATGR 265-5A (below)**.

As mentioned in previous section, attempts are being made at ICRISAT to introgress stress resistance through AB-breeding approach in grain legumes. In pigeonpea, ICRISAT is in the process of developing two backcross populations (ICPL 87119 × ICPW 29 and ICPL 87119 × ICPW 12) for conducting AB-QTL analysis and their subsequent use in AB-breeding. Among wild relatives used, ICPW 29 belongs to *C*. *cajanifolius* while ICPW 12 to *C. acutifolius* species. From the above crosses, BC_2_F_3_ seeds have been generated for multilocation phenotyping and selection. In groundnut, using two of the above mentioned synthetics, two AB-QTL mapping populations from the crosses between ICGV 91114 (cultivated) and ISATGR 1212 (*A. duranensis* ICG 8123 × *A. ipaënsis* ICG 8206, synthetic amphiploid); and between ICGV 87846 (cultivated) and ISATGR 265-5A (*A. kempff-mercadoi* ICG 8164 × *A. hoehnei* ICG 8190, synthetic amphiploid) have been developed at ICRISAT. Considerable variability has been observed in these two populations for pod and seed traits (Figure 3). A subset of these two populations has been phenotyped and genotyped with DArT markers to construct genetic maps and conducting AB-QTL analysis. The backcross (BC_1_F_1_ and BC_2_F_1_) lines carrying the alien genomic segments with maximum recurrent parent genomic regions provided optimal distribution of the synthetic genome introgressions (Foncéka et al., 2009).

In case of chickpea multi-parent advanced generation intercross (MAGIC) populations are being developed to enhance the genetic base. Eight elite lines/cultivars (ICC 4958, ICCV 10, JAKI 9218, JG 11, JG 130, JG 16, ICCV 97105, and ICCV 00108) were selected from Ethiopia, Kenya and India for development of a MAGIC population for *desi* chickpea. Twenty-eight two-way, 14 four-way and seven eight-way crosses were made to develop this MAGIC population. The best progenies from each generation are being used in various national breeding programs by the breeders. Currently, 1200 *F*_6_ are in the field (crop season 2012-2013). A total of 44 pre-breeding populations have also been developed.

The availability of large number of markers and cost-effective genotyping systems in recent years has ensured that linkage drag has been limited through stringent background selection and monitoring of the wild genomic regions. Hence, keeping in view the low genetic diversity in all the three legume crops, deployment of genomic-assisted pre-breeding is needed for accelerated and efficient enrichment of the cultivated gene pool. This should lead to the incorporation of desired/favorable alleles from wild relatives, increasing genetic gain and ultimately improving yield under adverse conditions and diverse cultural practices.

## Conclusions

For grain legume improvement, sufficient genetic diversity exists in the form of landraces and wild relatives, which carry several useful genes for cultivar improvement. However, utilization of these resources in breeding programs is time-consuming and resource-demanding. To overcome this, pre-breeding activities should be initiated to generate new genetic variability using promising landraces and wild relatives for use by the breeders in crop improvement programs. Pre-breeding should focus on the continuous supply of useful variability into the breeding pipeline to develop new high-yielding cultivars with a broad genetic base; pre-breeding should not focus on increasing yield. Though pre-breeding is useful to enrich the primary gene pool for cultivar improvement, it is a time-consuming and difficult affair as well. As mentioned by Nass and Paterniani (2000), promising results can only be obtained in 5–10 years depending upon the information available in the beginning of the program. Further, linkage drag associated with utilizing wild relatives makes the pre-breeding activities much more cumbersome. Genomic-assisted pre-breeding will help to overcome the linkage drag and will facilitate focused transfer of useful genes/segments from wild relatives for genetic enhancement of grain legumes.

### Conflict of interest statement

The authors declare that the research was conducted in the absence of any commercial or financial relationships that could be construed as a potential conflict of interest.
